# Principle of Least Effort and Sentence Length in Public Speaking

**DOI:** 10.3390/e23081023

**Published:** 2021-08-08

**Authors:** Natalia L. Tsizhmovska, Leonid M. Martyushev

**Affiliations:** 1 Technical Physics Department, Ural Federal University, 19 Mira St., 620002 Ekaterinburg, Russia; n.l.tsizhmovska@urfu.ru; 2 Institute of Industrial Ecology, Russian Academy of Sciences, 20 S. Kovalevskaya St., 620219 Ekaterinburg, Russia

**Keywords:** quantitative linguistics, sentence lengths, principle of least effort, Weibull distribution, minimum entropy production principle

## Abstract

The analysis of sentence lengths in the inaugural speeches of US presidents and the annual speeches of UK party leaders is carried out. Transcripts of the speeches are used, rather than the oral production. It is discovered that the average sentence length in these speeches decreases linearly with time, with the slope of 0.13 ± 0.03 words/year. It is shown that among the analyzed distributions (log-normal, folded and half normal, Weibull, generalized Pareto, Rayleigh) the Weibull is the best distribution for describing sentence length. These two results can be considered a consequence of the principle of least effort. The connection of this principle with the well-known principles of maximum and minimum entropy production is discussed.

## 1. Introduction

The study of natural languages is extremely important not only for the human and social sciences, but also for the sciences that study the development patterns of complex systems (synergetics, cybernetics, etc.). An important section of language science is quantitative linguistics, which uses mathematical methods to establish language laws (note that the objectives and methods of quantitative linguistics go beyond the mere study of linguistic laws, see, e.g., [1,2]). At present, several similar laws are considered, and among them, the most famous are Zipf’s law, Herdan’s law, Brevity law, and Menzerath–Altmann’s law [3,4,5,6,7]. Such laws, found mostly by statistical methods, indicate existing regularities between various elements of language (phonemes, words, etc.).

The most important element of language is the sentence—the object of this study. According to the Cambridge dictionary, a sentence is a group of words, usually containing a verb, that expresses a thought in the form of a statement, question, instruction, or exclamation. Sentences have semantic completeness; they express a particular thought of a person and serve to communicate it with other people. Based on the above sentence qualities, the study of these structural units is essential for cognitive science, which is of great interest. A metaphor from atomic physics would be very appropriate to illustrate this, especially for representatives of the natural sciences. Many properties of an atom are estimated by radiation (spontaneous and stimulated) that an atom emits and/or absorbs. A person (human brain) also “emits” and perceives elementary flows of thought in the form of sentences and the characteristics of this “human radiation” can reveal a lot about both the person and their environment.

An important quantitative characteristic of a sentence is its length, which can be measured in various ways (the number of letters, words, etc.). The study of sentence lengths does not require special linguistic training and can be easily processed by computer. As a result, this value has been studied for a long time and is used to determine the authorship of a work, the genre of the text, the cognitive development of the author or reader (listener), the level of language proficiency, etc. [8,9,10,11,12,13,14]. Two regularities are noticed regarding sentence length.

The first regularity is a decrease in the average sentence length over time. The decrease may vary depending on the genre and language of the text [15,16,17,18]. In particular, according to analysis of English texts [15]: “fiction sentences are approximately (on average) 6.5 words shorter now than they were in the beginning of the nineteenth century”. The second regularity is the asymmetry of sentence length distribution in the text (their distribution functions are not normal). Various laws are proposed to describe sentence length distribution; log-normal is the most often, but it is also suggested to use others, in particular, gamma and hyperpascal distributions [4,6,19,20,21,22,23,24]. These regularities are associated with various factors; in particular, attempts are made to connect the log-normal distribution law with some stochastic multiplicative processes of sentence formation and the central limit theorem in logarithmic space [22]. There is no single general explanation of the noted regularities to date.

At the same time, the so-called principle of least effort [25] has existed for a long time in cognitive linguistics. According to this principle, language changes because speakers simplify their speech in various ways. This principle was suggested by G. Zipf. In 1949 he wrote: “a person, in solving his immediate problems, will view these against the background of his future problems, as estimated by himself. Moreover, he will strive to solve his problems in such a way as to minimize the total work that he must expend in solving both his immediate problems and his probable future problems. That in turn means that the person will strive to minimize the probable average rate of his work-expenditure (over time). And in so doing he will be minimizing his effort. Least effort, therefore, is a variant of least work.” [25]. Note that G. Zipf is not the first to consider this kind of principle. In discussing the close connection between thinking and language, it is necessary to mention E. Mach and his principle of the economy of thought (1864): “when the human mind, with its limited powers, attempts to mirror in itself the rich life of the world, of which it itself is only a small part, and which it can never hope to exhaust, it has every reason for proceeding economically” [26].

Starting with G. Zipf, the discussed principle of least effort is used to explain the different frequencies of words of various lengths, the origins of scaling in human language, etc. (see, e.g., [27,28]). However, even at the sentence level, this principle from a single position allows us to explain the two above-mentioned regularities. In fact, languages have evolved so that language users can communicate using sentences that are relatively easy to produce and comprehend. It is worth quoting a fragment from Ref. [29] “Various models of human sentence production and comprehension predict that long dependencies are difficult or inefficient to process; minimizing dependency length thus enables effective communication without incurring processing difficulty”. Thus, with a long-term observation of the language, sentence length will decrease. Let us consider the application of this principle for a significantly smaller timescale—creation time of the text by the author. The author strives to express each of his thoughts in the most economical, shortest way. As a result, the author consciously and unconsciously tends to use sentences of the minimum length (*L*), among the variety of those that are similar in content {*L_1_, L_2_, …, L_n_*}, i.e., *L* = min{*L_1_, L_2_, …, L_n_*}. It is well known from mathematical statistics [30,31] that the distribution of the minima of a random variable corresponds to the Weibull distribution (strictly, if *L* = min{*L_1_, L_2_, …, L_n_*}, *n*→∞ and, *L_1_, L_2_, …, L_n_* being identically distributed random variables equal to zero or larger, *L* will obey the Weibull distribution function [30,31]). Thus, the principle of least effort unambiguously indicates that sentence lengths, with a sufficiently large sample, should be described by the Weibull distribution, and not by any other distributions. It is interesting to note that the Weibull distribution is a two-parameter asymmetric distribution that generalizes the well-known one-parameter Rayleigh distribution and can be reduced to a gamma distribution by changing the variable.

The purpose of this work is to check the applicability of the Weibull distribution to the distribution of sentence lengths and to discover the law of the sentences length decrease over time. The results can provide additional justification for the applicability of the principle of least effort to elementary units of human speech that carry a particular thought.

## 2. Data for Analysis

The object of this research was to study the public speeches of politicians. Previously, this has beencarried out several times (see, e.g., [32,33,34]). However, the objectives of those studies were different from the objective of this work (readability and sentiment analysis, letter frequency distribution, etc.). Political speeches are a convenient object of research, since this is a form of oral speech that is well-documented for sufficiently long times. Political speeches are positioned between spoken and written ways of expressing thoughts. Unlike spoken speech, the speech under consideration is more meaningful, prepared ahead of time, and less spontaneous, from the speaker’s point of view. At the same time, in comparison with written speech, political speeches are more focused on the listener, and, therefore, have a greater emotional component and the tendency to be easily understood. As a result, political speeches are extremely valuable research material. Such speeches are usually focused on some “average” citizen—the voter—therefore, the processing of such data reflects the temporal changes in the majority of native speakers.

We analyzed text transcripts of the 59 inaugural speeches of US presidents from 1789 to 2021 and 224 texts of speeches of UK Party leaders from 1895 to 2018 (available in [35] and [36], respectively). The studied speeches of US presidents are uniformly distributed every four years. The time distribution of speeches of UK Party leaders was not so uniform (due to copyright, the appearance of a new large party in parliament in 1977, etc.), but much more extensive. Note that no UK speeches were processed for 1898, 1914–1917, 1931, 1938–1940, 1944, 1952–1954, or1959. The list of analyzed speeches is presented in Appendixes.

**Figure 1:**
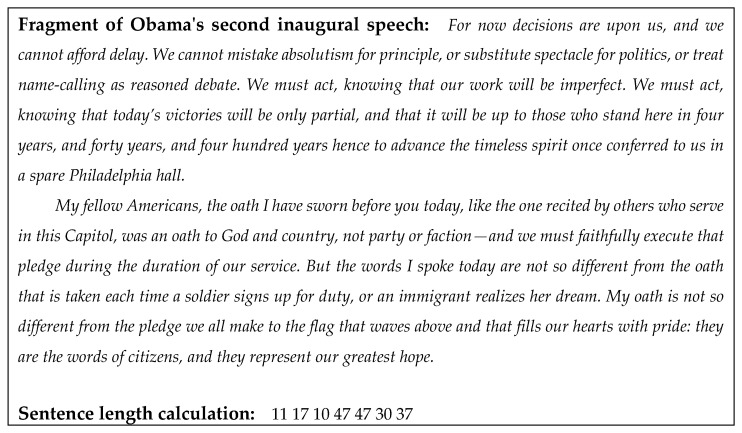
Example of sentence length calculation by the algorithm used in the study.

Sentence length was calculated from period to period, the unit of measurement was the words between spaces (prior to analysis, we replace all question marks, exclamation marks, and ellipses with a period, and also remove all dots used when writing decimal numbers). Note that the selected unit of measure for sentence length is not exclusive. Words were selected as a unit of measure for sentence length primarily because of the simplicity and the great prevalence of this approach. It is necessary to note that according to [37], sentence length is robust with respect to the selection of the unit of measurement. Thus, the choice of the word (and, e.g., not letters) will not lead to a change in the results of further analysis. The calculation was carried out automatically using a developed and tested computer program (see, example in Figure 1).

**Figure 2:**
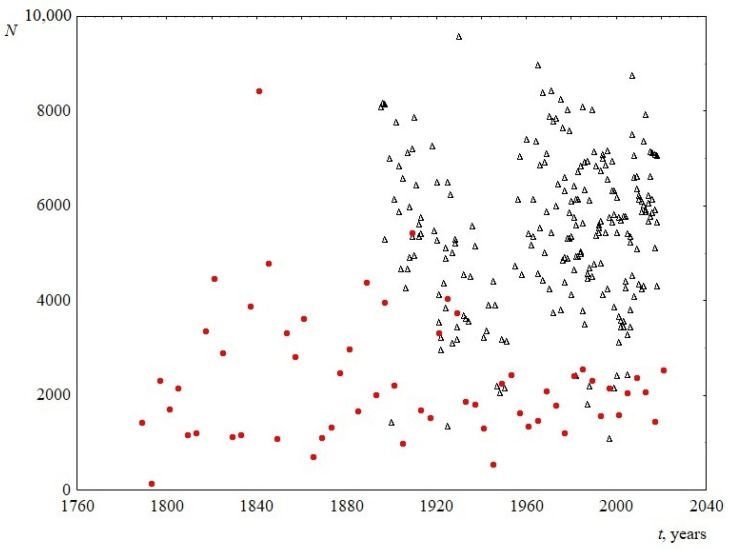
Number of words in the text *N* versus time *t*. Red circles indicate data for USA, and black triangles indicate data for UK.

Despite the fact that the studied speeches belonged to a long period of time, the total number of words in the speeches did not change reliably (Figure 2). The average length of speech in words was 2331 ± 355 for the US and 5434 ± 774 for the UK.

Statistical analysis was performed using the well-known and widespread professional commercial product Statistica 12.0 (TIBCO Software). The data (year of the speech and values corresponding to the processed sentence lengths of speeches) are in open access [38]. 

## 3. Change in Sentence Length over Time

The parameters characterizing sentence length were calculated. They are listed below.

**Figure 3:**
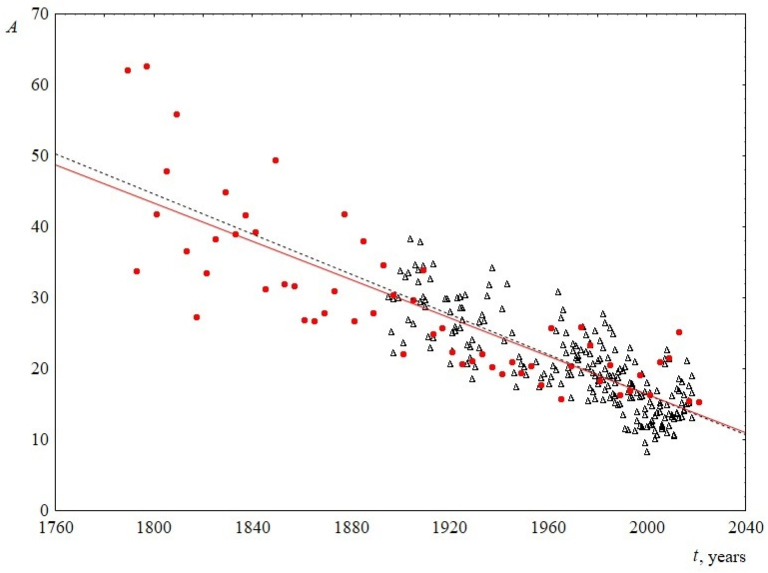
Average sentence length ***A*** as a function of the time speaking ***t***. Red circles indicate data for USA, and black triangles indicate data for UK. The linear regression equation for USA is 286–0.13***t*** and for UK is 299–0.14***t***.

**Figure 4:**
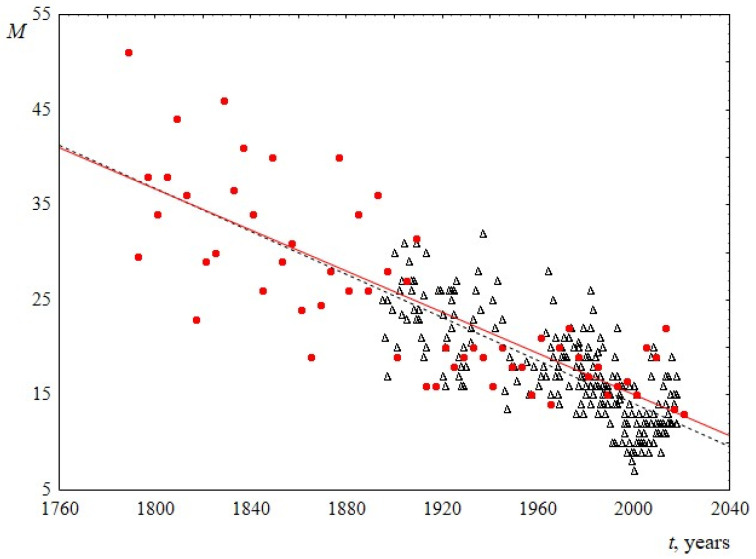
Median *M* as a function of the time speaking *t*. Red circles indicate data for USA, and black triangles indicate data for UK. The linear regression equation for USA is 232–0.11*t* and for UK is 240–0.11*t*.

**Figure 5:**
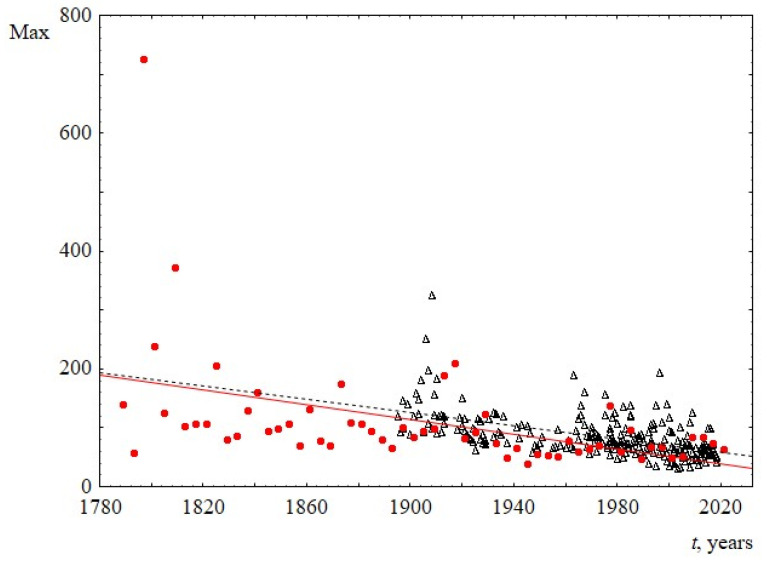
Maximum sentence length Max, as a function of the time speaking t. Red circles indicate data for USA, and black triangles indicate data for UK. The linear regression equation for USA is 1317–0.6*t* with the slope of 0.6 ± 0.3. The linear regression equation for UK is 1197–0.6*t* with the slope of 0.6 ± 0.1.

1. The average sentence length. To calculate this parameter, the total number of words in a speech was divided by the number of sentences. The change in this parameter over time is shown in Figure 3. The figure demonstrates that the average sentence length decreases linearly, with the slopes for USA and UK practically coinciding, and are equal to 0.13 ± 0.03 and 0.14 ± 0.01, respectively. On average, over 100 years, from 1900 to 2000, the average sentence length for both sets decreases from 30 to 16 words in a sentence, that is, the length is reduced by almost twice.

2. The median is known to be a stable characteristic of the distribution, it is almost unaffected by outliers. According to Figure 4, the median sentence length distribution decreases linearly in both sets with time. The slopes of the lines for USA and UK are 0.11 ± 0.02 and 0.11 ± 0.01, respectively. It can be seen that the lines are very close and practically coincide with ones for average sentence lengths.

3. The decrease in sentence length over time is also demonstrated in Figure 5, where the time dependence of the maximum sentence length is presented. The decrease in this parameter in both sets is approximately linear.

The final results of this section are summarized in Table 1.

## 4. Analysis of the Sentence Length Distribution Law

To analyze the sentence length distribution law, a number of speeches of the US presidents were excluded from the initial data. First, small speech texts, containing less than 40 sentences were excluded (these are speech texts of 1789, 1793, 1797, 1813, 1829, 1833, 1849, 1865, 1869, 1905, and 1945). Second, since it is the texts of public oral speeches that are analyzed, the texts of 1953, 1961, 1973, and 1981 were excluded because these speeches were not spoken, but were only written. Third, speech texts of 1801, 1805, 1837, 1877, 1881, 1893, 1941, 1965, and 1969 were not processed, since these speech texts have a multimodal distribution (the reasons for this and the analysis of these distributions could be the subject of a separate work). Thus, the analysis of the distribution law was carried out at 31 inaugural speeches of US presidents. All 224 speeches of the UK party leaders were analyzed. However, 31 texts were excluded due to the low significance level (<0.05) of the results obtained in relation to all tested distribution laws. Single outliers were excluded from the datasets before data analysis.

Six distributions with no more than two parameters, such as log-normal, Weibull, folded normal, half normal (normal), generalized Pareto, Rayleigh were analyzed in order to find the best theoretical distribution that describes the studied empirical distributions. The ranking of these distributions by the quality of data description was carried out according to the Kolmogorov–Smirnov criterion: the larger the ***p***-level value, the better this distribution describes the empirical data and, accordingly, the higher its place in comparison with others. Table 2 and Table 3 show the number of times one of the six listed sentence length distributions was among the top three (see Appendix A for more information).

Table 2 shows that, in 14 inaugural speeches of the US presidents, the Weibull distribution took first place in terms of significance, in another 14 it took second place, and in 3, it took third place. Thus, the Weibull distribution is the only distribution that adequately describes all speeches and takes the top three places. The average distribution significance level, where Weibull was in the first place, is 0.73, and for the second and third places, it is 0.5 and 0.3, respectively (see Appendix A). The log-normal distribution, ranked in the top three for 19 speeches, describes the data somewhat worse than Weibull one. The average significance levels for the log-normal distribution are 0.67 for the first place (13 speeches), 0.37 for the second place (5 speeches) and 0.08 for the third place (1 speech). Similar results can be seen for UK speeches (see Table 3 and Appendix A).

**Figure 6:**
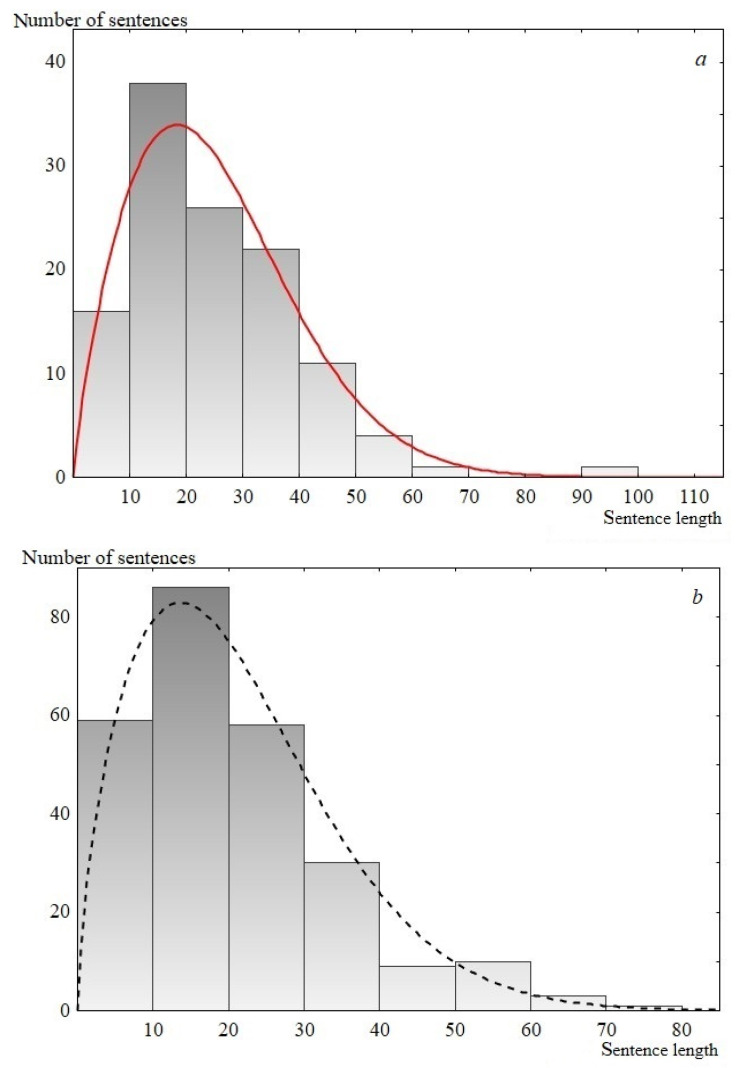
Sentence length distribution histogram. (**a**) USA speech, 1817; 119 points. The solid line is the Weibull distribution, for which *р*-level is 0.80, *λ* = 28.4 and *k* = 1.8; (**b**) UK Labour Party, 1992; 256 points. The dashed line is the Weibull distribution, for which р-level is 0.42, *λ* = 23.8 and *k* = 1.7.

Thus, according to the performed statistical analysis, the Weibull distribution is the most preferable for describing the studied speeches. The Weibull distribution (cumulative distribution function) has the form 1 − exp(− (x/λ)^k^), where λ and k are the scale and shape parameters respectively. Examples of the experimental data description using the Weibull distribution are presented in Figure 6. Note that the one-parameter Rayleigh distribution, ranked third in the description quality according to the analysis results, is a special case of the Weibull distribution, where the shape parameter is equal to two (see Table 2 and Table 3).

**Figure 7:**
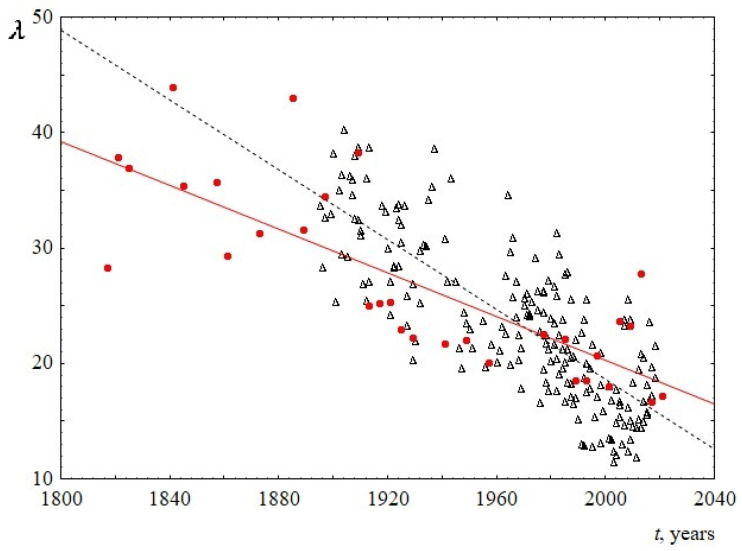
Behavior of the scale parameter *λ* of the Weibull distribution versus the time *t*. Red circles indicate data for USA, and black triangles indicate data for UK. The linear regression equation for USA is 209.8–0.09*t* with the slope of 0.09 ± 0.03. The linear regression equation for UK is 321.5–0.15*t* with the slope of 0.15 ± 0.02.

**Figure 8:**
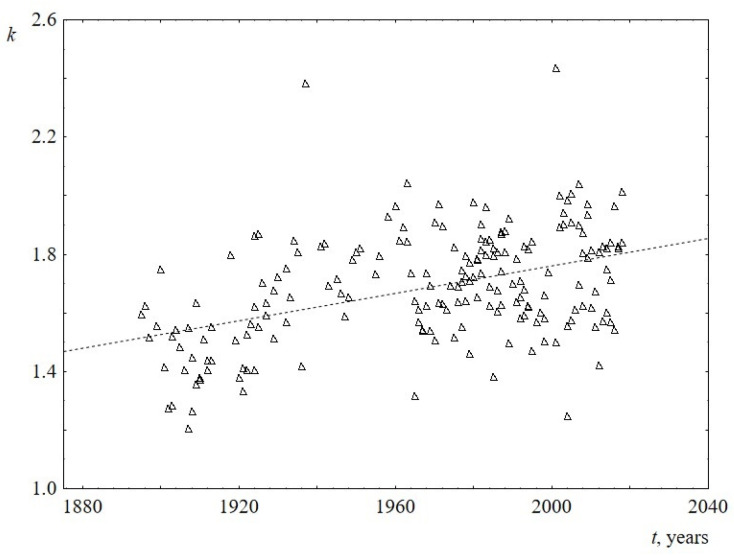
Behavior of the shape parameter *k* of the Weibull distribution versus the time *t* for UK. The linear regression equation is -2.9 + 0.002*t* (the slope of the line is 0.002 ± 0.001).

The behavior of the parameters of the Weibull distribution over time is shown in Figure 7 and Figure 8 (see Appendix B for numerical details). It follows from the Figure 7 that the scale parameter reliably decreases over time. Since this parameter is known to be directly proportional to the mean, median, and mode of the Weibull distribution, this once again confirms the above statement about the decrease in the average (and the most probable) sentence length. The shape parameter for US speeches does not reliably change over time and is equal to 1.9 ± 0.1. At the same time, the shape parameter for UK speeches is slightly increasing, changing from 1.5 to 1.8 over the past 100 years. This is the only difference found when comparing sentence lengths for US and UK speeches. Since the change over time is not large (the slope of the line is 0.002 ± 0.001), for reliability, an analysis of this result using additional data is required. Table 4 summarizes the results on the behavior of the parameters of the Weibull distribution over time.

**Figure 9:**
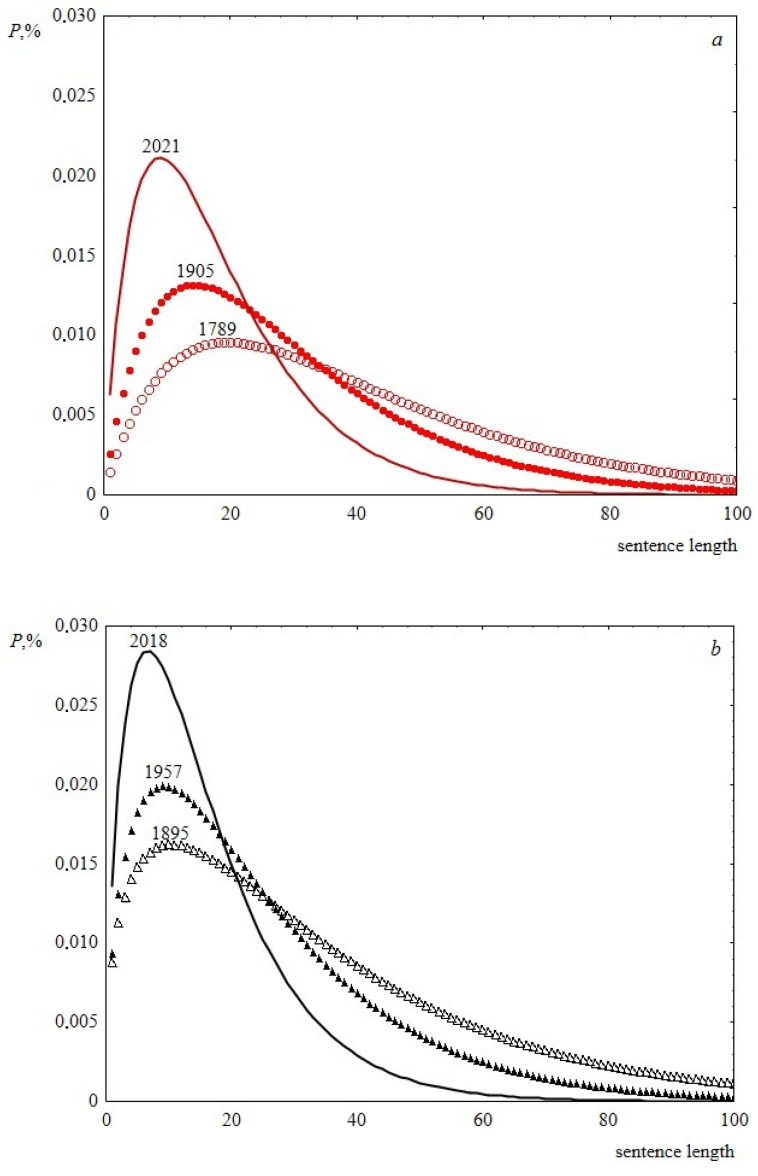
Weibull distribution histograms showing the change in sentence length distributions over time. (**a**) Data for USA, the shape parameter is 1.9, the scale parameters are 40.2, 29.2 and 18.2 for 1789, 1905 and 2021, respectively. (**b**) Data for UK, the shape parameters are 1.4, 1.6, 1.7 and the scale parameters are 34.6, 25.2 and 15.9 for 1895, 1957 and 2018, respectively.

**Figure 10:**
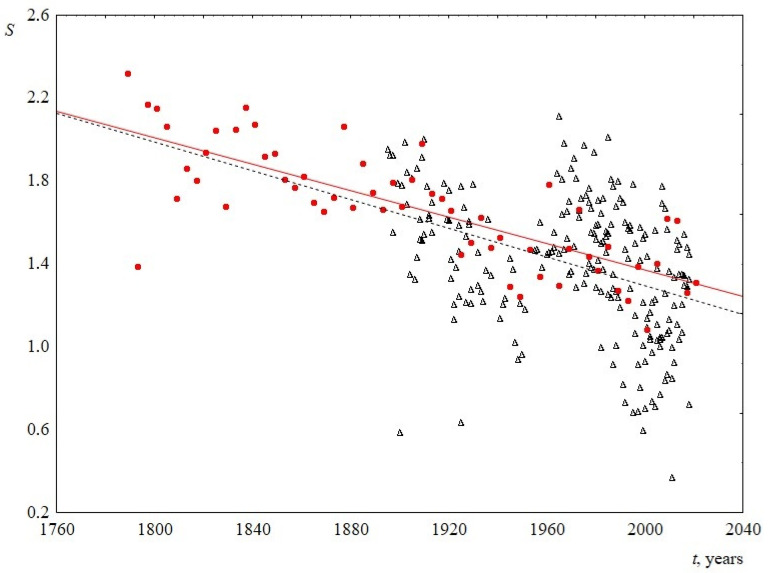
Information entropy S versus the time *t*. Red circles indicate data for USA, and black triangles indicate data for UK. The linear regression equation for USA is 7.7–0.003*t* (the slope of the line is 0.003 ± 0.001) and for UK is 8.2–0.003*t* (the slope of the line is 0.003 ± 0.001).

Thus, the time behavior of the parameters of the Weibull distribution allows us to conclude that, over the past two hundred years, sentence length distribution has become less fuzzy, the width of the peak decreases, and its abscissa is slightly shifted to the left. This is demonstrated in Figure 9. As a result, over time, speeches become composed of similar in length and shorter sentences, the difference in length decreases. In terms of sentence lengths, the text becomes more ordered. This can be seen in Figure 10, where information entropy (Shannon entropy) is presented as a function of time. The calculation of this value was based on sentence length distribution histograms containing the probabilities of detecting sentence length in a speech at a certain length interval.

## 5. Conclusions

Based on the calculation of sentence lengths in the text transcripts of the inaugural speeches of the US presidents for 228 years and the annual speeches of the UK party leaders for 123 years, two main results were obtained:

1. The average sentence length for both US and UK speeches decreases linearly with time with the slope of 0.13 ± 0.03 words/year and, on average, from 1900 to 2000, sentence length decreased with time from 30 to 16 words.

2. Sentence length distribution for both US and UK speeches is better described by the Weibull distribution (in particular, in comparison with the log-normal). The scale parameter of this distribution reliably decreases over time from 35 to 15. The shape parameter for US speeches does not change over time and is equal to 1.9 ± 0.1, and the shape parameter for UК speeches slightly changes over time from 1.5 to 1.8.

These two results are in agreement with the principle of least effort: the speaker, attempting to minimize both their efforts and the listeners’ effort, tends to choose the shortest possible sentence length from a potential set of sentences of approximately the same content. As a result, on the one hand, sentence length distribution begins to correspond to the distribution of minimum values—the Weibull distribution, and on the other hand, at time intervals significantly longer than the speech preparation time, the average sentence length decreases. The detected change over time in the scale parameter of the Weibull distribution and in information entropy indicates that sentence length in public speeches is gradually becoming less diverse; it is being unified and standardized. 

Here we highlight the following idea. When establishing the distribution type for empirical data, most important are not statistical tests, but rather the theoretical justification. If we accept the principle of least effort, then the Weibull distribution clearly follows from it. If one assumes that the principle of least effort is not suitable here, then obviously, they must propose some other theoretical justification—their principle—and theoretically derive, for example, gamma or lognormal distributions from it. Currently, we do not see such attempts. The G. Zipf’s principle, in our opinion, is very profound and productive, and many interesting consequences can be obtained from it. It has great potential, which has not yet been fully embraced by modern linguists. Our work and a number of works (see, e.g., [27,28,39,40,41,42,43]) show how useful it can be.

An interesting continuation of this work can be the verification of the obtained results using breath groups [6]. Detecting correlations and differences in such a collaborative analysis of breath groups (largely related to human physiology) and sentence lengths (largely related to cognitive processes) is a very interesting task. One of the problems in this direction will be a significantly smaller statistical database for breath groups in comparison with an almost limitless database for sentence lengths. Another interesting development of this work, in the scope of currently well-established directions connected to language complex networks (see, e.g., [39,40,41,42,43]), could be an analysis of the data obtained, here, from the position of the principle of compression, which appeared as a development of the ideas of G. Zipf [43]. It seems to us that the results of this work, combined with the principle of compression and with the use of Kolmogorov complexity ideas (existing inalgorithmic information theory) could be very promising. Found patterns for English also require validation for other languages, including artificial, as well as using other methods and linguistic units (letters, initial characters, words, etc.). In this regard, works [44,45,46] may be useful.

In conclusion, we return to the metaphor from physics given in the introduction. The analysis of the atom emission spectra and the Planck formula for wavelength distribution revolutionized the understanding of atomic properties, leading to the formulation of the laws of the quantum world—quantum mechanics. The distribution law of the lengths of utterances (sentences) “emitted” by the brain, corresponding to the Weibull distribution, is also able to stimulate the development of brain sciences. One of the possible directions related to brain biophysics may be the study of the energetic basis of the origin and development of thought and language. There are a number of works in this direction, in particular [4,6,47,48,49,50,51,52,53]. Considering thought as a complex non-equilibrium process, it can be concluded that its development matches the well-known principle of maximum entropy production. According to this principle, causes (stimuli) generate such responses that maximize the thermodynamic entropy production [47,54,55]. One of these responses in the course of the evolution of human thinking was the origin of the language. This revolutionary bifurcation process led to an abrupt increase in energy consumption and, as a result, an increase in the entropy production in a nonequilibrium system, i.e., in neural networks of humans who have become users of language. Naturally, a spontaneously emerged structure (network) could not be optimal at inception: only a certain basic structure (framework) of language was formed, which had some imperfections. Subsequently, being already at the high level of energy consumption and entropy production achieved after bifurcation, the nonequilibrium system began to evolve for a rather long time, trying to minimize energy consumption [54,55]. This minimization will no longer return the system to its previous, pre-bifurcation values of entropy production; however, due to the optimization of the neural network processes responsible for language, a small decrease is possible. According to nonequilibrium thermodynamics, this optimization process is already progressing in accordance with the Prigogine minimum production principle [47,54,55]. Its linguistic analogue can be considered the principle of least effort (least effort assumes less energy spent on communication, and, consequently, less energy dissipation). The information on the “simplification” of language—a decrease in its entropy, discovered in this work, can be considered a confirmation that language is currently going through a second (minimizing) stage of development.

## Figures and Tables

**Table 1:** Time behavior of the average sentence length.

Parameters Characterizing Sentence Length	Linear Fitting, Confidence Level, Coefficient of Determination	
	USA	UK
Average	286 − (0.13 ± 0.03)*t*, 95%, 0.67	299 – (0.14 ± 0.01)*t*, 95%, 0.64
Median	232 – (0.11 ± 0.02)*t*, 95%, 0.64	240 – (0.11 ± 0.01)*t*, 95%, 0.57
Maximum value	1317 – (0.6 ± 0.3)*t*, 95%, 0.19	1197 – (0.6 ± 0.1)*t*, 95%, 0.29

**Table 2:** Ranking of distributions according to the Kolmogorov–Smirnov criterion. US speeches.

		Weibull	Log-Normal	Rayleigh	Folded Normal	Normal	General Pareto
Place	1	14	13	3	1	0	0
	2	14	5	6	4	1	0
	3	3	1	9	8	8	1
∑		31	19	18	13	9	1

**Table 3:** Ranking of distributions according to the Kolmogorov–Smirnov criterion. UK speeches.

		Weibull	Log-Normal	Rayleigh	Folded Normal	Normal	General Pareto
Place	1	144	62	4	2	1	1
	2	47	43	27	23	8	0
	3	2	20	14	16	19	0
∑		193	125	45	41	28	1

**Table 4:** Parameters of the Weibull distribution for USA and UK speeches.

Parameters of the Weibull Distribution	Linear Fitting, Confidence Level, Coefficient of Determination	
	USA	UK
Scale (*λ*)	209.8 − (0.09 ± 0.03)*t*, 95%, 0.63	321.5 − (0.15 ± 0.02)*t*, 95%, 0.64
Shape (*k*)	1.9 ± 0.1, 95%	−2.9 + (0.002 ± 0.001)*t*, 95%, 0.19

## Data Availability

Not applicable.

## Appendix A

Ranking the suitability of theoretical distribution laws for the observed empirical sentence length distributions based on the Kolmogorov–Smirnov criterion. After the name of the distribution, its *p*-level is given:

DATA FOR USA

**Year**	**I Place, *p*-Value**	**II Place, *p*-Value**	**III Place, *P*-Value**
1817	Weibull, 0.80	Log Normal, 0.52	Rayleigh, 0.33
1821	Log Normal, 0.51	Weibull, 0.41	Rayleigh, 0.20
1825	Log Normal, 0.97	Weibull, 0.79	Folded Normal, 0.56
1841	Log Normal, 0.98	Weibull, 0.38	Rayleigh, 0.10
1845	Log Normal, 0.72	Weibull, 0.30	Rayleigh, 0.02
1857	Folded Normal, 0.97	Weibull, 0.96	Normal, 0.90
1861	Weibull, 0.91	Log Normal, 0.45	Normal, 0.16
1873	Rayleigh, 0.86	Weibull, 0.85	Folded Normal, 0.62
1885	Weibull, 0.80	Folded Normal, 0.66	Normal, 0.59
1889	Rayleigh, 0.54	Weibull, 0.54	Folded Normal, 0.24
1897	Weibull, 0.57	Log Normal, 0.52	Rayleigh, 0.49
1909	Weibull, 0.97	Rayleigh, 0.72	Folded Normal, 0.68
1913	Log Normal, 0.54	Weibull, 0.18	Half Normal, 0.12
1917	Log Normal, 0.85	Weibull, 0.30	Half Normal, 0.12
1921	Weibull, 0.43	Log Normal, 0.18	Rayleigh, 0.06
1925	Rayleigh, 0.25	Weibull, 0.20	Log Normal, 0.08
1929	Weibull, 0.26	Rayleigh, 0.13	Folded Normal, 0.03
1941	Log Normal, 0.75	Weibull, 0.73	Folded Normal, 0.17
1949	Log Normal, 0.54	Weibull, 0.54	Folded Normal, 0.19
1957	Log Normal, 0.56	Rayleigh, 0.26	Weibull, 0.24
1977	Weibull, 0.90	Rayleigh, 0.75	Normal, 0.65
1985	Log Normal, 0.40	Weibull, 0.22	Rayleigh, 0.09
1989	Log Normal, 0.72	Rayleigh, 0.5	Weibull, 0.49
1993	Weibull, 0.89	Folded Normal, 0.59	Rayleigh, 0.54
1997	Log Normal, 0.41	Rayleigh, 0.20	Weibull, 0.18
2001	Weibull, 0.45	Log Normal, 0.19	Folded Normal, 0.18
2005	Weibull, 0.92	Folded Normal, 0.56	Normal, 0.53
2009	Weibull, 0.97	Half Normal, 0.23	General Pareto, 0.16
2013	Log Normal, 0.75	Weibull, 0.63	Rayleigh, 0.60
2017	Weibull, 0.71	Folded Normal, 0.51	Normal, 0.33
2021	Weibull, 0.67	Log Normal, 0.31	Folded Normal, 0.01

DATA FOR UK

**Year, Party**	**I Place, *p*-Value**	**II Place, *p*-Value**	**III Place, *p*-Value**
1895 Liberal	Log Normal, 0.37	Weibull, 0.37	Folded Normal, 0.01
1896 Liberal	Log Normal, 0.46	Weibull, 0.28	Normal, 0.00
1897 Liberal	Log Normal, 0.06	Weibull, 0.01	Half Normal, 0.00
1897 Conservative	Weibull, 0.47	Log Normal, 0.22	Half Normal, 0.02
1899 Liberal	Weibull, 0.29	Log Normal, 0.15	Normal, 0.00
1900 Conservative	Weibull, 0.87	Folded Normal, 0.64	Log Normal, 0.57
1901 Liberal	Weibull, 0.31	Log Normal, 0.07	Half Normal, 0.05
1902 Conservative	Folded Normal, 0.46	Half Normal, 0.46	Weibull, 0.30
1903 Conservative	Weibull, 0.84	Half Normal, 0.39	Log Normal, 0.26
1903 Liberal	Weibull, 0.51	Folded Normal, 0.08	Normal, 0.06
1904 Conservative	Weibull, 0.64	Folded Normal, 0.42	General Pareto, 0.18
1905 Liberal	Weibull, 0.18	Log Normal, 0.14	Half Normal, 0.01
1906 Conservative	Half Normal, 0.37	Weibull, 0.36	General Pareto, 0.23
1907 Conservative	General Pareto, 0.25	Weibull, 0.16	Log Normal, 0.13
1907 Liberal	Weibull, 0.53	Half Normal, 0.13	Normal, 0.09
1908 Liberal	Weibull, 0.59	Folded Normal, 0.13	Half Normal, 0.13
1908 Conservative	Weibull, 0.66	Log Normal, 0.45	Half Normal, 0.27
1909 Liberal	Weibull, 0.69	Folded Normal, 0.3	Log Normal, 0.05
1909 Conservative	Weibull, 0.34	Log Normal, 0.34	Half Normal, 0.16
1910 Liberal	Weibull, 0.38	Folded Normal, 0.27	Half Normal, 0.26
1910 Conservative	Weibull, 0.92	Half Normal, 0.24	Folded Normal, 0.24
1911 Conservative	Weibull, 0.72	Log Normal, 0.13	Folded Normal, 0.07
1912 Liberal	Weibull, 0.55	Log Normal, 0.3	Folded Normal, 0.21
1912 Conservative	Weibull, 0.17	Log Normal, 0.14	Half Normal, 0.03
1913 Conservative	Log Normal, 0.11	Weibull, 0.07	Half Normal, 0.02
1913 Liberal	Weibull, 0.91	Log Normal, 0.28	Folded Normal, 0.26
1918 Liberal	Weibull, 0.82	Rayleigh, 0.17	Log Normal, 0.07
1919 Liberal	Weibull, 0.98	Half Normal, 0.12	Log Normal, 0.09
1920 Liberal	Weibull, 0.66	Folded Normal, 0.65	Half Normal, 0.62
1920 Conservative	Weibull, 0.02	Log Normal, 0.02	Folded Normal, 0.01
1921 Liberal	Weibull, 0.67	Log Normal, 0.63	Half Normal, 0.23
1921 Conservative	Log Normal, 0.58	Weibull, 0.09	Half Normal, 0.07
1922 Liberal	Weibull, 0.90	Half Normal, 0.73	General Pareto, 0.51
1922 Conservative	Weibull, 0.52	Log Normal, 0.48	Folded Normal, 0.10
1923 Liberal	Weibull, 0.85	General Pareto, 0.25	Normal, 0.15
1924 Liberal	Log Normal, 0.85	Weibull, 0.50	Folded Normal, 0.26
1924 Labour	Weibull, 0.77	Half Normal, 0.24	Folded Normal, 0.24
1924 Conservative	Weibull, 0.75	Log Normal, 0.39	Folded Normal, 0.11
1925 Liberal	Log Normal, 0.83	Weibull, 0.60	Rayleigh, 0.33
1925 Conservative	Weibull, 0.47	Log Normal, 0.02	Normal, 0.02
1926 Conservative	Weibull, 0.21	Log Normal, 0.09	Rayleigh, 0.01
1927 Liberal	Log Normal, 0.67	Weibull, 0.21	Folded Normal, 0.01
1927 Conservative	Log Normal, 0.47	Weibull, 0.19	Folded Normal, 0.01
1928 Liberal	Log Normal, 0.35	Weibull, 0.04	Half Normal, 0.00
1928 Conservative	Log Normal, 0.45	Weibull, 0.01	Folded Normal, 0.00
1929 Liberal	Log Normal, 0.37	Weibull, 0.19	Folded Normal, 0.03
1929 Conservative	Log Normal, 0.67	Weibull, 0.61	Folded Normal, 0.05
1930 Liberal	Weibull, 0.16	Log Normal, 0.00	Normal, 0.00
1932 Conservative	Weibull, 0.78	Log Normal, 0.23	Normal, 0.10
1932 Liberal	Log Normal, 0.52	Weibull, 0.45	Folded Normal, 0.03
1933 Conservative	Log Normal, 0.70	Weibull, 0.57	Folded Normal, 0.08
1934 Conservative	Weibull, 0.75	Folded Normal, 0.72	Normal, 0.67
1935 Conservative	Weibull, 0.61	Folded Normal, 0.53	Rayleigh, 0.32
1936 Liberal	Log Normal, 0.89	Weibull, 0.19	Folded Normal, 0.03
1937 Liberal	Weibull, 0.74	Folded Normal, 0.46	Log Normal, 0.41
1941 Liberal	Weibull, 0.68	Log Normal, 0.54	Rayleigh, 0.43
1942 Liberal	Weibull, 0.67	Rayleigh, 0.38	Log Normal, 0.31
1943 Liberal	Log Normal, 0.74	Weibull, 0.51	Normal, 0.07
1945 Liberal	Weibull, 0.62	Rayleigh, 0.23	Normal, 0.11
1946 Labour	Log Normal, 0.29	Weibull, 0.08	Normal, 0.00
1947 Labour	Log Normal, 0.76	Weibull, 0.21	Normal, 0.01
1948 Labour	Log Normal, 0.85	Weibull, 0.51	Normal, 0.04
1949 Labour	Weibull, 0.47	Log Normal, 0.38	Folded Normal, 0.08
1950 Labour	Log Normal, 0.55	Weibull, 0.39	Normal, 0.11
1951 Labour	Log Normal, 0.85	Weibull, 0.19	Rayleigh, 0.02
1955 Conservative	Weibull, 0.32	Log Normal, 0.29	Rayleigh, 0.03
1956 Conservative	Weibull, 0.11	Log Normal, 0.07	Folded Normal, 0.01
1957 Conservative	Log Normal, 0.38	Weibull, 0.05	Folded Normal, 0.00
1958 Conservative	Log Normal, 0.15	Weibull, 0.13	Rayleigh, 0.09
1960 Conservative	Weibull, 0.53	Rayleigh, 0.35	Folded Normal, 0.03
1961 Conservative	Weibull, 0.68	Rayleigh, 0.1	Log Normal, 0.04
1962 Conservative	Weibull, 0.49	Rayleigh, 0.17	Log Normal, 0.17
1963 Liberal	Log Normal, 0.58	Weibull, 0.09	Rayleigh, 0.01
1963 Conservative	Log Normal, 0.52	Rayleigh, 0.43	Weibull, 0.32
1964 Labour	Weibull, 0.75	Folded Normal, 0.18	Log Normal, 0.08
1965 Labour	Log Normal, 0.1	Weibull, 0.09	General Pareto, 0.02
1965 Conservative	Log Normal, 0.49	Weibull, 0.25	Normal, 0.00
1966 Labour	Weibull, 0.46	Folded Normal, 0.06	Log Normal, 0.02
1966 Conservative	Weibull, 0.32	Log Normal, 0.19	Folded Normal, 0.02
1967 Labour	Weibull, 0.66	Folded Normal, 0.04	Log Normal, 0.01
1967 Conservative	Log Normal, 0.36	Weibull, 0.13	Half Normal, 0.00
1968 Labour	Weibull, 0.73	Folded Normal, 0.07	Log Normal, 0.01
1968 Conservative	Log Normal, 0.22	Weibull, 0.16	Rayleigh, 0.00
1969 Labour	Weibull, 0.55	Folded Normal, 0.03	Normal, 0.00
1969 Conservative	Weibull, 0.18	Log Normal, 0.05	Half Normal, 0.00
1970 Labour	Weibull, 0.28	Log Normal, 0.03	Folded Normal, 0.01
1970 Conservative	Log Normal, 0.41	Weibull, 0.35	Rayleigh, 0.16
1971 Conservative	Log Normal, 0.21	Weibull, 0.15	Rayleigh, 0.1
1971 Labour	Weibull, 0.43	Folded Normal, 0.05	Normal, 0.01
1972 Conservative	Log Normal, 0.37	Weibull, 0.21	Folded Normal, 0.07
1972 Labour	Weibull, 0.66	Log Normal, 0.06	Normal, 0.00
1973 Conservative	Log Normal, 0.17	Weibull, 0.01	Folded Normal, 0.00
1973 Labour	Weibull, 0.57	Folded Normal, 0.1	Normal, 0.04
1974 Labour	Weibull, 0.81	Log Normal, 0.02	Normal, 0.01
1975 Conservative	Log Normal, 0.53	Weibull, 0.28	Rayleigh, 0.06
1975 Labour	Weibull, 0.41	Folded Normal, 0.04	Log Normal, 0.02
1976 Conservative	Weibull, 0.23	Log Normal, 0.07	Normal, 0.00
1976 Labour	Weibull, 0.24	Log Normal, 0.05	Normal, 0.00
1977 Conservative	Weibull, 0.48	Normal, 0.03	Rayleigh, 0.03
1997 Labour	Weibull, 0.54	Log Normal, 0.03	Folded Normal, 0.01
1977 Liberal a	Weibull, 0.66	Log Normal, 0.10	Rayleigh, 0.02
1977l Liberal b	Log Normal, 0.67	Weibull, 0.3	Folded Normal, 0.01
1978 Conservative	Weibull, 0.26	Log Normal, 0.04	Folded Normal, 0.01
1978 Labour	Weibull, 0.19	Log Normal, 0.01	Normal, 0.00
1978 Liberal	Weibull, 0.28	Log Normal, 0.12	Folded Normal, 0.05
1979 Conservative	Log Normal, 0.30	Weibull, 0.12	Folded Normal, 0.00
1979 Labour	Weibull, 0.17	Log Normal, 0.02	Folded Normal, 0.00
1989 Liberal	Weibull, 0.58	Rayleigh, 0.03	Normal, 0.02
1980 Conservative	Weibull, 0.45	Rayleigh, 0.37	Log Normal, 0.17
1980 Labour	Log Normal, 0.14	Weibull, 0.02	Folded Normal, 0.00
1980 Liberal	Weibull, 0.63	Log Normal, 0.11	Rayleigh, 0.02
1981 Conservative	Weibull, 0.69	Rayleigh, 0.08	Log Normal, 0.08
1981 Labour	Log Normal, 0.51	Weibull, 0.20	Half Normal, 0.00
1981 Liberal	Weibull, 0.31	Log Normal, 0.19	Folded Normal, 0.02
1982 SDP-Liberal Alliance b	Weibull, 0.69	Folded Normal, 0.34	Rayleigh, 0.34
1982 Conservative	Weibull, 0.18	Rayleigh, 0.05	Log Normal, 0.05
1982 Labour	Weibull, 0.96	Rayleigh, 0.26	Normal, 0.15
1982 Liberal	Weibull, 0.24	Log Normal, 0.05	Rayleigh, 0.01
1983 Conservative	Weibull, 0.17	Log Normal, 0.13	Rayleigh, 0.06
1983 Labour	Weibull, 0.57	Folded Normal, 0.1	Rayleigh, 0.08
1983 Liberal	Weibull, 0.51	Rayleigh, 0.41	Folded Normal, 0.05
1984 Conservative	Weibull, 0.81	Folded Normal, 0.21	Normal, 0.02
1984 Labour	Weibull, 0.60	Normal, 0.05	Folded Normal, 0.04
1984 Liberal	Weibull, 0.07	Rayleigh, 0.02	Log Normal, 0.00
1985 Conservative	Weibull, 0.23	Log Normal, 0.10	Rayleigh, 0.05
1985 Labour	Log Normal, 0.42	Weibull, 0.29	Half Normal, 0.08
1985 Liberal	Weibull, 0.63	Log Normal, 0.08	Rayleigh, 0.05
1986 Conservative	Weibull, 0.35	Log Normal, 0.09	Folded Normal, 0.07
1986 Labour	Weibull, 0.64	Log Normal, 0.04	Normal, 0.01
1986 Liberal	Weibull, 0.28	Log Normal, 0.01	Folded Normal, 0.01
1987 Conservative	Weibull, 0.51	Rayleigh, 0.09	Log Normal, 0.02
1987 Labour	Weibull, 0.21	Log Normal, 0.06	Folded Normal, 0.01
1987 SDP-Liberal Alliance a	Weibull, 0.61	Log Normal, 0.25	Folded Normal, 0.15
1987 SDP-Liberal Alliance b	Log Normal, 0.37	Weibull, 0.16	Rayleigh, 0.03
1988 Conservative	Weibull, 0.47	Rayleigh, 0.09	Folded Normal, 0.03
1988 Labour	Log Normal, 0.22	Weibull, 0.03	Half Normal, 0.00
1988 Liberal	Log Normal, 0.9	Weibull, 0.42	Rayleigh, 0.07
1989 Conservative	Weibull, 0.12	Rayleigh, 0.05	Log Normal, 0.01
1989 Labour	Log Normal, 0.09	Weibull, 0.06	Half Normal, 0.00
1990 Conservative	Weibull, 0.16	Log Normal, 0.02	Folded Normal, 0.00
1990 Labour	Log Normal, 0.24	Weibull, 0.02	Half Normal, 0.00
1991 Conservative	Weibull, 0.11	Log Normal, 0.01	Rayleigh, 0.00
1991 Labour	Weibull, 0.11	Log Normal, 0.04	Normal, 0.00
1992 Conservative	Weibull, 0.26	Folded Normal, 0.00	Log Normal, 0.00
1992 Labour	Weibull, 0.42	Log Normal, 0.05	Rayleigh, 0.00
1992 Liberal Democrat l	Weibull, 0.39	Log Normal, 0.18	Normal, 0.00
1993 Conservative	Weibull, 0.23	Log Normal, 0.02	Folded Normal, 0.00
1993 Labour	Weibull, 0.51	Rayleigh, 0.22	Normal, 0.09
1993 Liberal Democrat	Folded Normal, 0.51	Weibull, 0.43	Normal, 0.10
1994 Conservative	Weibull, 0.11	Log Normal, 0.05	Normal, 0.00
1994 Labour	Weibull, 0.36	Log Normal, 0.01	Normal, 0.00
1994 Liberal Democrat	Weibull, 0.88	Folded Normal, 0.17	Rayleigh, 0.14
1995 Conservative	Weibull, 0.17	Rayleigh, 0.01	Log Normal, 0.00
1995 Labour	Log Normal, 0.25	Weibull, 0.12	Half Normal, 0.00
1996 Conservative	Log Normal, 0.14	Weibull, 0.03	Folded Normal, 0.00
1996 Labour	Log Normal, 0.26	Weibull, 0.03	Folded Normal, 0.00
1996 Liberal Democrat	Weibull, 0.44	Log Normal, 0.003	Normal, 0.00
1997 Conservative	Weibull, 0.44	Folded Normal, 0.33	Log Normal, 0.31
1997 Labour	Log Normal, 0.12	Weibull, 0.001	Half Normal, 0.00
1998 Conservative	Weibull, 0.07	Log Normal, 0.05	Folded Normal, 0.00
1998 Labour	Log Normal, 0.19	Weibull, 0.13	Folded Normal, 0.00
1988 Liberal Democrat	Weibull, 0.09	Log Normal, 0.001	Folded Normal, 0.00
1999 Conservative	Log Normal, 0.07	Weibull, 0.04	Folded Normal, 0.00
1999 Labour	Log Normal, 0.01	Half Normal, 0.00	Weibull, 0.00
1999 Liberal Democrat a	Weibull, 0.74	Folded Normal, 0.28	Log Normal, 0.15
1999 Liberal Democrat b	Log Normal, 0.04	Weibull, 0.00	Rayleigh, 0.00
2000 Conservative	Weibull, 0.01	Log Normal, 0.01	Normal, 0.00
2000 Labour	Log Normal, 0.02	Weibull, 0.00	Folded Normal, 0.00
2000 L	Log Normal, 0.01	Weibull, 0.00	Half Normal, 0.00
2001 Conservative	Log Normal, 0.17	Weibull, 0.1	Folded Normal, 0.02
2001 Labour	Log Normal, 0.52	Weibull, 0.00	Half Normal, 0.00
2001 Liberal Democrat	Weibull, 0.13	Log Normal, 0.06	Half Normal, 0.00
2002 Conservative	Weibull, 0.28	Rayleigh, 0.27	Log Normal, 0.14
2002 Labour	Log Normal, 0.10	Weibull, 0.00	Folded Normal, 0.00
2002 Liberal Democrat	Weibull, 0.21	Rayleigh, 0.05	Folded Normal, 0.03
2003 Conservative	Weibull, 0.16	Rayleigh, 0.11	Folded Normal, 0.01
2003 Labour	Log Normal, 0.20	Weibull, 0.02	Half Normal, 0.00
2003 Liberal Democrat	Weibull, 0.05	Rayleigh, 0.03	Folded Normal, 0.00
2004 Conservative	Weibull, 0.08	Rayleigh, 0.07	Folded Normal, 0.00
2004 Labour	Weibull, 0.24	Log Normal, 0.18	Half Normal, 0.01
2004 Liberal Democrat l	Weibull, 0.19	Folded Normal, 0.01	Log Normal, 0.00
2005 Conservative	Weibull, 0.64	Rayleigh, 0.30	Log Normal, 0.14
2005 Labour	Weibull, 0.07	Log Normal, 0.05	Folded Normal, 0.00
2005 Liberal Democrat	Rayleigh, 0.20	Weibull, 0.19	Log Normal, 0.11
2006 Conservative a	Weibull, 0.15	Log Normal, 0.05	Folded Normal, 0.00
2006 Conservative b	Weibull, 0.01	Log Normal, 0.00	Rayleigh, 0.00
2006 Labour	Log Normal, 0.12	Weibull, 0.03	Folded Normal, 0.00
2006 Liberal Democrat	Log Normal, 0.24	Weibull, 0.03	Rayleigh, 0.01
2007 Conservative	Weibull, 0.10	Log Normal, 0.09	Folded Normal, 0.00
2007 Labour	Weibull, 0.16	Rayleigh, 0.02	Log Normal, 0.02
2007 Liberal Democrat	Rayleigh, 0.19	Weibull, 0.11	Log Normal, 0.05
2008 Conservative	Weibull, 0.17	Log Normal, 0.03	Normal, 0.00
2008 Labour	Weibull, 0.19	Log Normal, 0.06	Rayleigh, 0.01
2008 Liberal Democrat l	Weibull, 0.49	Rayleigh, 0.07	Normal, 0.01
2009 Conservative	Weibull, 0.06	Log Normal, 0.01	Rayleigh, 0.00
2009 Labour	Rayleigh, 0.68	Weibull, 0.53	Folded Normal, 0.1
2009 Liberal Democrat	Weibull, 0.17	Rayleigh, 0.05	Folded Normal, 0.01
2010 Conservative	Weibull, 0.23	Log Normal, 0.00	Folded Normal, 0.00
2010 Labour	Weibull, 0.45	Folded Normal, 0.04	Rayleigh, 0.02
2010 Liberal Democrat l	Log Normal, 0.16	Weibull, 0.03	Rayleigh, 0.01
2011 Conservative	Weibull, 0.30	Rayleigh, 0.0003	Log Normal, 0.00
2011 Labour	Log Normal, 0.02	Weibull, 0.01	Normal, 0.00
2011 Liberal Democrat	Weibull, 0.25	Folded Normal, 0.00	Log Normal, 0.00
2012 Conservative	Weibull, 0.16	Folded Normal, 0.02	Half Normal, 0.01
2012 Labour	Weibull, 0.01	Log Normal, 0.01	Folded Normal, 0.00
2012 Liberal Democrat	Weibull, 0.49	Log Normal, 0.13	Rayleigh, 0.03
2013 Conservative	Weibull, 0.19	Log Normal, 0.03	Folded Normal, 0.00
2013 Labour	Log Normal, 0.11	Weibull, 0.02	Normal, 0.00
2013 Liberal Democrat	Weibull, 0.64	Rayleigh, 0.11	Normal, 0.03
2014 Conservative	Weibull, 0.13	Log Normal, 0	Folded Normal, 0.00
2014 Labour	Weibull, 0.17	Folded Normal, 0.01	Rayleigh, 0.00
2014 Liberal Democrat	Weibull, 0.32	Folded Normal, 0.13	Normal, 0.03
2015 Conservative	Weibull, 0.2	Folded Normal, 0.001	Log Normal, 0.00
2015 Labour	Weibull, 0.18	Log Normal, 0.01	Rayleigh, 0.00
2015 Liberal Democrat	Weibull, 0.42	Folded Normal, 0.07	Rayleigh, 0.03
2016 Conservative	Log Normal, 0.23	Weibull, 0.01	Normal, 0.00
2016 Labour	Weibull, 0.37	Rayleigh, 0.31	Log Normal, 0.12
2016 Liberal Democrat	Log Normal, 0.14	Weibull, 0.07	Normal, 0.00
2017 Conservative	Log Normal, 0.05	Weibull, 0.02	Normal, 0.00
2017 Labour	Weibull, 0.16	Log Normal, 0.1	Folded Normal, 0.01
2017 Liberal Democrat	Weibull, 0.31	Rayleigh, 0.11	Folded Normal, 0.04
2018 Conservative	Log Normal, 0.12	Rayleigh, 0.01	Weibull, 0.003
2018 Labour	Rayleigh, 0.18	Weibull, 0.16	Log Normal, 0.09
2018 Liberal Democrat	Weibull, 0.41	Rayleigh, 0.1	Log Normal, 0.09

## Appendix B

The shape and scale parameters for the Weibull distributions used for the description of the empirical sentence length distributions

DATA FOR USA

**Year**	***p*-Value**	λ**, Scale**	***k*, Shape**
1817	0.80	28.4	1.8
1821	0.41	37.9	1.9
1825	0.79	36.9	1.9
1841	0.38	43.9	1.9
1845	0.30	35.4	1.8
1857	0.96	35.8	2.3
1861	0.91	29.3	1.7
1873	0.85	31.3	2.0
1885	0.80	43.0	2.2
1889	0.54	31.6	2.0
1897	0.57	34.4	2.0
1909	0.97	38.3	1.9
1913	0.18	25.0	1.4
1917	0.30	25.3	1.5
1921	0.43	25.3	1.7
1925	0.20	23.0	2.0
1929	0.26	22.2	1.9
1941	0.73	21.7	1.6
1949	0.54	22.0	2.4
1957	0.24	20.1	2.0
1977	0.90	22.6	2.3
1985	0.22	22.1	1.9
1989	0.49	18.5	2.0
1993	0.89	18.5	2.3
1997	0.18	20.7	2.0
2001	0.45	18.0	2.5
2005	0.92	23.7	2.3
2009	0.97	23.3	1.5
2013	0.63	27.8	2.3
2017	0.71	16.7	1.7
2021	0.67	17.2	1.6

DATA FOR UK

**Year, Party**	***p*-Value**	λ**, Scale**	***k,* Shape**
1895 Liberal	0.37	33.7	1.6
1896 Liberal	0.28	28.3	1.6
1897 Conservative	0.47	32.6	1.5
1899 Liberal	0.29	32.9	1.6
1900 Conservative	0.87	38.2	1.7
1901 Liberal	0.31	25.3	1.4
1902 Conservative	0.30	35.0	1.3
1903 Conservative	0.84	36.4	1.3
1903 Liberal	0.51	29.5	1.5
1904 Conservative	0.64	40.2	1.5
1905 Liberal	0.18	29.3	1.5
1906 Conservative	0.36	36.2	1.4
1907 Conservative	0.16	34.6	1.2
1907 Liberal	0.53	35.9	1.5
1908 Liberal	0.59	32.5	1.4
1908 Conservative	0.66	38.0	1.3
1909 Liberal	0.69	38.7	1.6
1909 Conservative	0.34	32.4	1.4
1910 Liberal	0.38	31.5	1.4
1910 Conservative	0.92	31.1	1.4
1911 Conservative	0.72	26.9	1.5
1912 Liberal	0.55	36.0	1.4
1912 Conservative	0.17	25.4	1.4
1913 Conservative	0.07	27.0	1.4
1913 Liberal	0.91	38.7	1.5
1918 Liberal	0.82	33.6	1.8
1919 Liberal	0.98	33.2	1.5
1920 Liberal	0.66	30.0	1.4
1921 Liberal	0.67	27.1	1.4
1921 Conservative	0.09	24.2	1.3
1922 Liberal	0.90	28.4	1.4
1922 Conservative	0.52	28.3	1.5
1923 Liberal	0.85	33.4	1.6
1924 Liberal	0.50	32.4	1.9
1924 Labour	0.77	28.4	1.4
1924 Conservative	0.75	33.8	1.6
1925 Liberal	0.60	30.4	1.9
1925 Conservative	0.47	32.0	1.6
1926 Conservative	0.21	33.7	1.7
1927 Liberal	0.21	23.3	1.6
1927 Conservative	0.19	25.9	1.6
1929 Liberal	0.19	20.3	1.7
1929 Conservative	0.61	26.9	1.5
1930 Liberal	0.16	22.0	1.7
1932 Conservative	0.78	25.2	1.8
1932 Liberal	0.45	29.7	1.6
1933 Conservative	0.57	30.3	1.7
1934 Conservative	0.75	30.1	1.8
1935 Conservative	0.61	34.2	1.8
1936 Liberal	0.19	35.2	1.4
1937 Liberal	0.74	38.6	2.4
1941 Liberal	0.68	30.8	1.8
1942 Liberal	0.67	27.0	1.8
1943 Liberal	0.51	36.0	1.7
1945 Liberal	0.62	27.1	1.7
1946 Labour	0.08	21.3	1.7
1947 Labour	0.21	19.6	1.6
1948 Labour	0.51	24.4	1.7
1949 Labour	0.47	23.5	1.8
1950 Labour	0.39	23.0	1.8
1951 Labour	0.19	21.3	1.8
1955 Conservative	0.32	23.7	1.7
1956 Conservative	0.11	19.7	1.8
1958 Conservative	0.13	21.7	1.9
1960 Conservative	0.53	20.1	2
1961 Conservative	0.68	21.1	1.8
1962 Conservative	0.49	23.1	1.9
1963 Liberal	0.09	22.6	1.8
1963 Conservative	0.32	27.5	2.0
1964 Labour	0.75	34.6	1.7
1965 Labour	0.09	29.6	1.3
1965 Conservative	0.25	19.9	1.6
1966 Labour	0.46	30.9	1.6
1966 Conservative	0.32	25.8	1.6
1967 Labour	0.66	27.0	1.5
1967 Conservative	0.13	24.0	1.5
1968 Labour	0.73	22.4	1.6
1968 Conservative	0.16	20.3	1.7
1969 Labour	0.55	17.8	1.7
1969 Conservative	0.18	21.3	1.5
1970 Labour	0.28	25.0	1.5
1970 Conservative	0.35	25.6	1.9
1971 Conservative	0.15	24.1	2.0
1971 Labour	0.43	26.1	1.6
1972 Conservative	0.21	24.1	1.9
1972 Labour	0.66	24.3	1.6
1973 Labour	0.57	25.3	1.6
1974 Labour	0.81	29.1	1.7
1975 Conservative	0.28	24.6	1.8
1975 Labour	0.41	26.2	1.5
1976 Conservative	0.23	16.6	1.6
1976 Labour	0.24	22.8	1.7
1977 Conservative	0.48	19.5	1.7
1997 Labour	0.54	24.4	1.6
1977 Liberal a	0.66	26.3	1.7
1977l Liberal b	0.3	26.1	1.7
1978 Conservative	0.26	18.4	1.7
1978 Labour	0.19	21.8	1.6
1978 Liberal	0.28	22.4	1.8
1979 Conservative	0.12	17.6	1.7
1979 Labour	0.17	27.1	1.5
1989 Liberal	0.58	21.2	1.8
1980 Conservative	0.45	20.2	2.0
1980 Liberal	0.63	23.8	1.7
1981 Conservative	0.69	21.6	1.8
1981 Labour	0.2	26.7	1.7
1981 Liberal	0.31	23.5	1.8
1982 SDP-Liberal Alliance b	0.69	25.9	1.9
1982 Conservative	0.18	17.6	1.9
1982 Labour	0.96	31.3	1.8
1982 Liberal	0.24	20.4	1.7
1983 Conservative	0.17	19.1	1.8
1983 Labour	0.57	29.5	1.8
1983 Liberal	0.51	22.4	2.0
1984 Conservative	0.81	19.5	1.7
1984 Labour	0.6	23.8	1.6
1984 Liberal	0.07	21.2	1.8
1985 Conservative	0.23	16.7	1.8
1985 Labour	0.29	27.7	1.4
1985 Liberal	0.63	20.1	1.8
1986 Conservative	0.35	18.3	1.8
1986 Labour	0.64	27.9	1.6
1986 Liberal	0.28	21.2	1.7
1987 Conservative	0.51	17.1	1.9
1987 Labour	0.21	25.5	1.6
1987 SDP-Liberal Alliance a	0.61	18.2	1.7
1987 SDP-Liberal Alliance b	0.16	20.5	1.9
1988 Conservative	0.47	16.5	1.9
1988 Liberal	0.42	20.6	1.8
1989 Conservative	0.12	16.9	1.9
1989 Labour	0.06	22.0	1.5
1990 Conservative	0.16	15.1	1.7
1991 Conservative	0.11	13.0	1.8
1991 Labour	0.11	22.7	1.6
1992 Conservative	0.26	12.8	1.7
1992 Labour	0.42	23.8	1.7
1992 Liberal Democrat	0.39	18.8	1.6
1993 Conservative	0.23	17.5	1.6
1993 Labour	0.51	25.6	1.8
1993 Liberal Democrat	0.43	19.9	1.7
1994 Conservative	0.11	19.6	1.6
1994 Labour	0.36	19.6	1.6
1994 Liberal Democrat	0.88	17.8	1.8
1995 Conservative	0.17	12.7	1.8
1995 Labour	0.12	21.7	1.5
1996 Liberal Democrat	0.44	15.3	1.6
1997 Conservative	0.44	17.1	1.6
1998 Conservative	0.07	18.1	1.7
1998 Labour	0.13	20.9	1.6
1988 Liberal Democrat	0.09	13.1	1.5
1999 Liberal Democrat a	0.74	15.9	1.7
2001 Conservative	0.1	18.0	2.4
2001 Liberal Democrat	0.13	13.4	1.5
2002 Conservative	0.28	16.8	2.0
2002 Liberal Democrat	0.21	13.4	1.9
2003 Conservative	0.16	11.4	1.9
2003 Liberal Democrat	0.05	12.4	1.9
2004 Conservative	0.08	12.0	2.0
2004 Labour	0.24	17.7	1.2
2004 Liberal Democrat	0.19	14.8	1.6
2005 Conservative	0.64	16.7	1.9
2005 Labour	0.07	16.4	1.6
2005 Liberal Democrat	0.19	15.3	2.0
2006 Conservative a	0.15	13.0	1.6
2007 Conservative	0.1	23.2	1.7
2007 Labour	0.16	23.8	1.9
2007 Liberal Democrat	0.11	14.7	2.0
2008 Conservative	0.17	16.2	1.6
2008 Labour	0.19	25.6	1.8
2008 Liberal Democrat	0.49	12.3	1.9
2009 Conservative	0.06	15.1	1.8
2009 Labour	0.53	23.8	2.0
2009 Liberal Democrat	0.17	13.4	1.9
2010 Conservative	0.23	14.5	1.6
2010 Labour	0.45	18.3	1.8
2011 Conservative	0.3	14.5	1.7
2011 Liberal Democrat	0.25	11.9	1.6
2012 Conservative	0.16	15.2	1.4
2012 Liberal Democrat	0.49	19.4	1.8
2013 Conservative	0.19	14.4	1.6
2013 Liberal Democrat	0.64	20.8	1.8
2014 Conservative	0.13	16.7	1.6
2014 Labour	0.17	14.9	1.8
2014 Liberal Democrat	0.32	20.5	1.7
2015 Conservative	0.2	15.5	1.6
2015 Labour	0.18	15.7	1.7
2015 Liberal Democrat	0.42	18.1	1.8
2016 Labour	0.37	23.6	2.0
2016 Liberal Democrat	0.07	16.8	1.5
2017 Labour	0.16	19.7	1.8
2017 Liberal Democrat	0.31	17.2	1.8
2018 Labour	0.16	21.5	2.0
2018 Liberal Democrat	0.41	18.7	1.8
